# Dietary polyphenols as a safe and novel intervention for modulating pain associated with intervertebral disc degeneration in an *in-vivo* rat model

**DOI:** 10.1371/journal.pone.0223435

**Published:** 2019-10-02

**Authors:** Alon Lai, Lap Ho, Thomas W. Evashwick-Rogler, Hironobu Watanabe, Jonathan Salandra, Beth A. Winkelstein, Damien Laudier, Andrew C. Hecht, Giulio M. Pasinetti, James C. Iatridis

**Affiliations:** 1 Leni & Peter W. May Department of Orthopaedics, Icahn School of Medicine at Mount Sinai, New York, New York, United States of America; 2 Department of Neurology, Icahn School of Medicine at Mount Sinai, New York, New York, United States of America; 3 James J. Peters Veterans Affairs Medical Center, Bronx, New York, United States of America; 4 Keiyu Orthopedic Hospital, Tatebayashi, Japan; 5 Philadelphia College of Osteopathic Medicine, Philadelphia, Pennsylvania, United States of America; 6 Department of Bioengineering, University of Pennsylvania, Philadelphia, Pennsylvania, United States of America; Zhejiang University, CHINA

## Abstract

Developing effective therapies for back pain associated with intervertebral disc (IVD) degeneration is a research priority since it is a major socioeconomic burden and current conservative and surgical treatments have limited success. Polyphenols are naturally occurring compounds in plant-derived foods and beverages, and evidence suggests dietary supplementation with select polyphenol preparations can modulate diverse neurological and painful disorders. This study tested whether supplementation with a select standardized Bioactive-Dietary-Polyphenol-Preparation (BDPP) may alleviate pain symptoms associated with IVD degeneration. Painful IVD degeneration was surgically induced in skeletally-mature rats by intradiscal saline injection into three consecutive lumbar IVDs. Injured rats were given normal or BDPP-supplemented drinking water. *In-vivo* hindpaw mechanical allodynia and IVD height were assessed weekly for 6 weeks following injury. Spinal column, dorsal-root-ganglion (DRG) and serum were collected at 1 and 6 weeks post-operative (post-op) for analyses of IVD-related mechanical and biological pathogenic processes. Dietary BDPP significantly alleviated the typical behavioral sensitivity associated with surgical procedures and IVD degeneration, but did not modulate IVD degeneration nor changes of pro-inflammatory cytokine levels in IVD. Gene expression analyses suggested BDPP might have an immunomodulatory effect in attenuating the expression of pro-inflammatory cytokines in DRGs. This study supports the idea that dietary supplementation with BDPP has potential to alleviate IVD degeneration-related pain, and further investigations are warranted to identify the mechanisms of action of dietary BDPP.

## Introduction

Low back pain affects 70–85% of the population at some time in their life and is the leading cause of disability worldwide [1]. While the cause of chronic back pain is multifactorial, the degree of lumbar intervertebral disc (IVD) degeneration is a main risk factor for non-specific back pain [2]. Degenerated IVDs are characterized by height loss, structural failure, spinal instability, decreased glycosaminoglycan and water contents, upregulated intradiscal pro-inflammatory cytokine expression, cell senescence, and neurovascular ingrowth [3]. IVD-related pain can result from spinal cord or nerve compression arising from IVD height loss, foraminal stenosis, IVD bulging and herniation, spinal instability or chronic inflammation among other pathologic mechanisms [4]. However, because of the complex etiology of axial, or discogenic, back pain, and challenges treating chronic conditions, there is little consensus on the best course of therapy [5]. Neither conservative therapies nor spinal fusion surgery result in significant improvement for IVD degeneration-related back pain [6–8]. While many challenges and opportunities exist for IVD repair there is much data to suggest that treatments for chronic back pain conditions might also require interventions that promote resilience and influence other spinal structures [8, 9]. There remains an urgent need to develop safe, minimally invasive and effective treatments for alleviating discogenic back pain.

Polyphenols are naturally occurring compounds that are widely distributed in many plant-derived foods and beverages, and dietary consumption of certain polyphenol-rich products have been shown to be safe and beneficial for a broad range of diseases, including cardiovascular disease and neurological disorders [10–12]. Health benefits of polyphenols are generally attributed to their anti-oxidant and anti-inflammatory characteristics [12–15], and more recent studies suggested polyphenol compounds also interfere with select disease-specific pathogenic mechanisms [16, 17]. Recent *in-vitro* evidence indicates select polyphenols can suppress pro-inflammatory mediators and matrix degrading enzymes, and reduce cell apoptosis [18–20], suggesting they may offer promise to modulate IVD degeneration and IVD-related pain. Local application of resveratrol or epigallocatechin 3-gallate (EGCG) was also shown to reduce painful radiculopathy in a surgically-induced IVD herniation model *in-vivo* [18, 19]. However, the effect of dietary polyphenols on IVD degeneration-related back pain is unclear.

**Fig 1:**
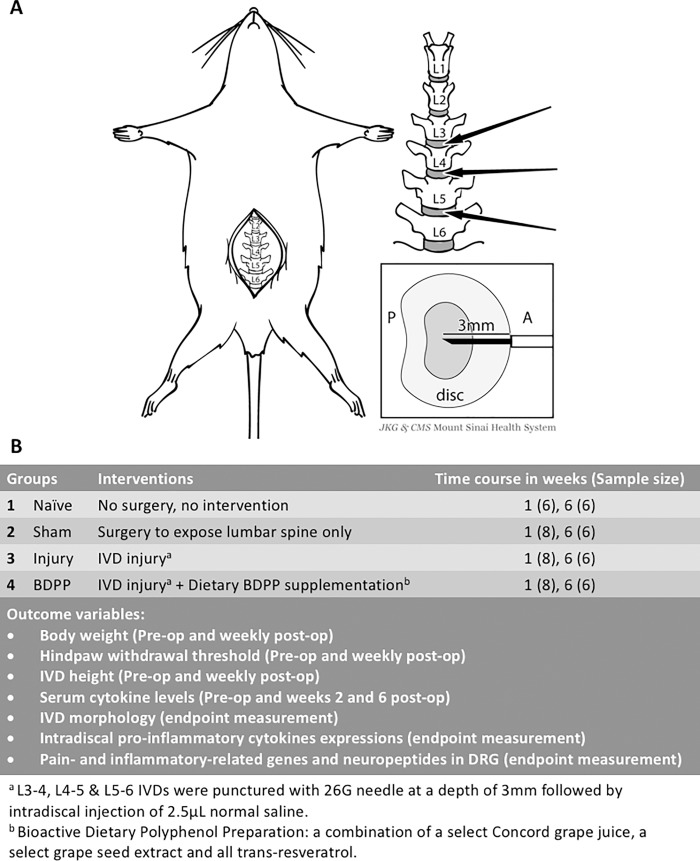
IVD injury and study design. (A) Schematic diagram illustrates the surgical procedure for inducing IVD injury. ‘P’ and ‘A’ represents posterior side and anterior side of IVD, respectively. After exposing the lumbar spine, the L3-4, L4-5 and L5-6 IVDs were punctured anteriorly along the midline to a depth of 3mm followed by intradiscal injection of normal saline. Image created from Mount Sinai Health System. Used with permission. (B) Experimental design of current study.

Development of *in-vivo* IVD degeneration models with painful behavioral phenotypes provide the necessary tools for mechanistic investigations on the pathophysiology of painful IVD degeneration and for screening potential therapeutic interventions. Rats are a widely accepted model for studying mechanisms of IVD degeneration because of structural similarities in spinal anatomy with the human spine [21]. Rats are also widely applied for pain studies since known quantitative methods exist to characterize their behavioral sensitivity [4, 22, 23]. We recently developed an *in-vivo* rat painful IVD degeneration model using anterior annular puncture of three adjacent lumbar IVDs followed by intradiscal saline injection (Fig 1A) [22, 23]. Animals exhibited painful behavior with moderate degeneration in the injured IVDs that are characterized by decreased magnetic-resonance-imaging signal intensity, decreased IVD height, disorganized annulus fibrosus (AF), fibrotic nucleus pulposus (NP), and reduced glycosaminoglycan content [22, 23]. Pain sensitivity was associated with IVD degeneration, IVD height loss, and inflammation similar to the human condition, and painful behavior was reduced by suppressing the pro-inflammatory cytokine response at the time of the acute injury [24]. However, despite this evidence, treatment strategies that are easily translatable to human are still needed for this painful condition.

We recently reported a non-intrusive intervention involving chronic dietary supplementation with a standardized, bioactive, polyphenol-rich dietary preparation, which we referred to as Bioactive-Dietary-Polyphenol-Preparation (BDPP). BDPP is a combination of bioactive and commercially available polyphenol product [16, 25, 26], and has significantly attenuated development of neuropathologic phenotypes and cognitive decline in diverse animal models of neurodegenerative disorders [16, 17, 27, 28]. Our pharmacokinetics and tissue accumulation studies have identified a large and diverse panel of BDPP-derived polyphenol metabolites that are biologically available *in-vivo* in the periphery and/or the brain, and therefore, are candidates to modulate biological processes. Moreover, our mechanistic studies revealed the BDPP dietary supplementation is effective in modulating inflammation and other neuropathogenic mechanisms [16, 17, 27, 28]. Overall, we proposed that BDPP supplementation may provide a potential safe and non-intrusive intervention for promoting resilience against IVD degeneration-related pain by suppressing the expression of pain-related mediators in IVDs and/or DRGs. This study aimed to test whether dietary BDPP supplementation alleviates IVD degeneration and -related pain in our *in-vivo* painful IVD degeneration rat model.

## Materials and methods

### Study design

Sixty-two skeletally mature male Sprague-Dawley rats (4–5 month-old) were randomly assigned to Naïve, Sham, Injury, or BDPP groups (Fig 1B). IVD degeneration was surgically-induced in rat lumbar IVDs in both Injury and BDPP groups. Naïve animals received no surgical procedures, while Sham rats received surgery to expose lumbar IVDs without any IVD puncture or injection. BDPP was provided to animals in the BDPP group via the drinking water, while animals in Naïve, Sham and Injury groups received normal drinking water. All animals have *ad libitum* access to food and their assigned drink.

Body weight (BW), hindpaw mechanical allodynia and IVD height was monitored at baseline (prior to surgery), and post-operative (post-op) weekly until week 6. Serum specimens were collected at 2-week and 6-week post-op. Moreover, at 1-week and 6-weeks post-op, subsets of animals were terminated, and their IVDs and DRGs were harvested for biological analyses. All experimental procedures were approved by the Institutional Animal Care and Use Committee at Icahn School of Medicine at Mount Sinai (Protocol number: LA13-00071). All rat studies were conducted in accordance with the U.S. National Institutes of Health Guidelines for the Care and Use of Experimental Animals, using protocols approved by the Institutional Animal Care and Use Committee of the Icahn School of Medicine at Mount Sinai.

### BDPP administration

BDPP provides a total of 509 mg polyphenol/kg of BW per day, with 29.5%, 12.2%, and 58.3% polyphenol composition from a select grape seed polyphenol extract, Welch Concord purple grape juice and all trans-resveratrol, respectively. The grape seed polyphenol extract and food grade all trans-resveratrol were purchased from supplement Warehouse (Universal Product Code: 603573579173) and ChromaDex (Irvine, CA), respectively. Welch Concord purple grape juice was purchased from local store. The polyphenol profile for each of the commercial products was validated by LCMS analysis and reported previously [29, 30]. A fresh stock of BDPP was prepared weekly and stored at 4°C in dark, and then diluted in drinking water. Consumption of BDPP-infused drinking water was documented and changed every 2–3 days.

### IVD injury procedure

IVD injury was performed under sterile conditions and general anesthesia (2% isoflurane in oxygen) as we have previously described [22]. In brief, rat lumbar spine was accessed using anterior approach with a midline abdominal incision. L3-4, L4-5 and L5-6 IVDs were identified using radiograph and anatomical landmarks of pelvic rim and aortic bifurcation. The three IVDs were sequentially punctured along their midline using a 26-gauge needle at a depth of 3 mm, guided by a needle stopper, followed by intradiscal injection of 2.5μL phosphate-buffered saline (PBS) using a calibrated micro-liter syringe (Fig 1A) [22]. The volume of 2.5ul and injection method was determined from our pilot in-vitro and in-vivo tests showing no visible fluid leakage, IVD swelling, or nerve root irritation [22, 23] (See Discussion). During the surgery, the 2.5ul of PBS was injected slowly over an injection duration of 10 seconds in order to minimize the possibility PBS leakage. The peritoneum and abdominal wall were closed using 3–0 silk sutures, and the skin was closed using 4–0 nylon sutures. After surgery, all rats were closely monitored to ensure no intraoperative complications after surgery.

### Assessment of behavioral sensitivity

Behavioral sensitivity, taken as a proxy of pain, of rat was assessed by measuring mechanical allodynia using the von Frey test applied to their hindpaws; this assay was shown to be a sensitive measurement for quantifying pain associated with IVD degeneration [22]. The von Frey test was performed in a wire-mesh floor cage. After 20 minutes of acclimation, calibrated von Frey filaments were applied to rat hindpaws to provide a calibrated force, ranging in ascending order from 0.4g to 26.0g, until a positive response was elicited [22, 23, 31, 32]. The 50% withdrawal threshold was determined using up-down method [33]. Withdrawal thresholds of both left and right hindpaws were determined with three trials for each side, and then averaged for analysis.

### Assessments of lumbar IVD height

Lumbar IVD height was quantified from lateral radiography under general anesthesia [22, 23, 34]. Radiographic films were digitized and magnified for measurement. Vertebral boundaries were identified manually, and IVD height was determined using a customized MATLAB script (Mathworks, Inc., Natick, MA) [22]. IVD heights from the injured IVDs were averaged for analysis.

### Assessments of IVD morphological degeneration

Subsets of animals were euthanized at weeks 1 and 6 post-op via carbon dioxide inhalation. Their lumbar spines (L2-4) were harvested, fixed, embedded in methyl methacrylate, and sectioned sagittally at 5μm intervals. Mid-sagittal sections were stained with safranin-O/light-green/hematoxylin for IVD morphology, and were evaluated using bright-field microscopy [22]. Severity of IVD degeneration was quantified using a semi-quantitative degeneration grading scale (Table 1) [24]. All sections were examined by two researchers blinded to the experimental groups, and then averaged for analysis.

**Table 1:** IVD degeneration grading scale. The scale is modified from previously developed and validated IVD degeneration grading schemes proposed by Masuda et al. [35] and Rutges et al. [36].

***Annulus fibrosus***		
**Grade**	**0**	Normal, pattern of fibrocartilage lamellae (U-shaped in posterior aspect and slightly convex in the anterior aspect) without ruptured fibers and without a serpentine appearance anywhere within the annulus
	**1**	Ruptured or serpentined pattern fibers in less than 30% of the annulus
	**2**	Ruptured or serpentined pattern fibers in greater than 30% of the annulus
***Border between annulus fibrosus and nucleus pulposus***		
**Grade**	**0**	Normal
	**1**	Minimally interrupted
	**2**	Moderate/severe interruption
***Cellularity of nucleus pulposus***		
**Grade**	**0**	Normal cellularity with large vacuoles in the gelatinous structure of the matrix
	**1**	Slight decrease in the number of cells and fewer vacuoles
	**2**	Moderate/severe decrease (>50%) in the number of cells and no vacuoles
***Matrix of nucleus pulposus***		
**Grade**	**0**	Normal gelatinous appearance
	**1**	Slight condensation of the extracellular matrix
	**2**	Moderate/severe condensation of the extracellular matrix
***Endplate***		
**Grade**	**0**	Homogenous structure; regular thickness
	**1**	Slight irregularity with limited number of microfractures; locally decreased thickness
	**2**	Severe irregularity with multiple microfractures of EP; generalized decrease of thickness

### Assessment of IVD inflammation

After euthanization, L4-5 IVD was isolated, snap-frozen in liquid nitrogen and stored at -80°C. Pro-inflammatory cytokines expressions were analyzed using western-blot. Total proteins were extracted from IVD tissue using tissue protein extraction reagent (Thermo Fisher Scientific, Waltham, MA) with protease inhibitor overnight at 4°C. 20μg of total protein from each IVD was loaded onto 12% polyacrylamide protein gel, electrophoresed, and then transferred to polyvinylidenefluoride membranes. After blocking in 5% nonfat milk, the membrane was incubated overnight at 4°C in 5% nonfat milk with rabbit anti-interleukin-1 beta (anti-IL-1β) (1:200 dilution, Bioss Antibodies Inc, Woburn, MA), mouse anti-IL-6 (1:1000 dilution, Abcam, Cambridge, MA), rabbit anti-tumor necrosis factor-alpha (anti-TNFα) (1:500 dilution, Novus Biologicals, Littleton, CO), or rabbit anti-GAPDH (1:10,000 dilution, Abcam) as internal control. After incubating in HRP-conjugated goat anti-rabbit IgG (1:3000 dilution, Bio-Rad Laboratories Inc.) or HRP-conjugated goat anti-mouse IgG (1:3000 dilution, Bio-Rad Laboratories Inc), the proteins were visualized by treating with enhanced chemiluminescence western blotting substrate (Pierce ECL Western Blotting Substrate, Thermo Fisher Scientific). The protein expressions were quantified using ImageJ, and normalized to GAPDH and Naïve for statistical analysis.

### Quantifications of pain- and inflammatory-related genes and neuropeptides in DRG

Bilateral L1-4 DRGs were resected following laminectomy for analyses since they were previously reported to innervate L3-6 IVDs [37, 38]. L2 DRGs were fixed for calcitonin gene-related peptide (CGRP) expression using immunohistochemistry; while L1, L3, and L4 DRGs were snap-frozen in liquid nitrogen and stored at -80°C for gene expression analyses using real time-quantitative PCR.

After fixation, the L2 DRGs were embedded and sectioned at 5μm intervals. Two non-serial sections were chosen from each animal. Following deparaffinization and protein blocking, the sections were incubated with either rabbit polyclonal primary antibody against rat CGRP (1:5000 dilution, Abcam) or normal rabbit serum (Biocare Medical, Concord, CA) as negative control. After incubation with horseradish peroxidase-conjugated anti-rabbit secondary antibody (ImmPRESS VR reagent, Vector Laboratories, Burlingame, CA) and 3% hydrogen peroxide, and the immunoreactivity was visualized using diaminobenzidine-based horseradish-peroxidase substrate (ImmPACT DAB, Vector Laboratories). The DRG sections were then counterstained with toluidine blue, dehydrated, mounted, and imaged at 20× magnification using bright-field light microscope with standardized exposure time. The small-sized (4–20 μm) and medium-sized (22–40 μm) DRG neurons [39, 40], corresponding to unmyelinated c-fibers and myelinated a-delta fibers respectively [41], were analyzed separately. The quantification method for CGRP immunopositive neurons was adopted and modified from the studies by Weisshaar et al. [39] and Dong et al. [40] in order to minimize the bias and subjective judgement in the analysis. In details, four subsections were randomly selected from each DRG section. In each subsection, twenty neurons with 10 small-sized and 10 medium-sized neurons were randomly selected, and therefore, a total of 80 neurons were obtained from each DRG section. The mean intensity and area of immunopositive staining for each neuron were measured. A threshold was assigned for both mean intensity (233) and percentage of area (20%), and any neuron that could meet the assigned threshold was classified as ‘immunopositive’. The percentage of small-sized and medium-sized immunopositive neurons from each DRG were determined and averaged with the non-serial section from the same animal for statistical analysis. All sections were examined by a researcher blinded to the experimental groups. The threshold values were identified from an experienced histologist after comparing more than 100 CGRP immunopositive neurons and negative control neurons. The results obtained from this quantification technique was highly repeatable with Intraclass correlation coefficient(3,1) of 0.974.

Gene expression analysis was conducted using real-time quantitative PCR (qPCR). Total RNA was extracted from DRGs using TRIzol reagent, purified and re-suspended in nuclease-free water, and then reverse-transcribed to cDNA according to manufacturer’s instructions. The reactions were carried out in a final volume of 25μl containing cDNA template, RT^2^ SYBR Green Mastermix (Qiagen), and forward and reverse rat-specific qPCR primers for each target gene (RT^2^ qPCR Primer Assay, Qiagen). The target genes included inflammatory cytokines (tnfα, il-1β, il-2, il-6, and il-10), chemoattractants (ccl2 and ccl12), and neurotransmitters and receptors (cgrp, tac1 and tacr1), and housekeeping gene GAPDH served as an internal control. Duplicate qPCR reactions were performed for each RNA sample and internal control. A negative control was also included using nuclease-free water instead of RNA sample. The qPCR was performed using Applied Biosystems 7500 real-time PCR System (Thermo Fisher Scientific). The threshold cycle (Ct) for each target gene was measured and normalized to GAPDH (ΔCt). The differences in ΔCt (ΔΔCt) and fold-differences (2^-ΔΔCt^) for Sham, Injury and BDPP groups relative to Naïve group were determined and compared between groups.

### Statistical analyses

BW, IVD height, and paw withdrawal threshold at each time point were normalized to baseline (pre-op), and the changes of normalized results with time within and across experimental groups were analyzed using two-way repeated measures ANOVA, followed by Tukey’s post-hoc comparison. Degeneration scores were compared across experimental groups using one-way ANOVA, followed by Tukey’s post-hoc comparison. Expressions of intradiscal pro-inflammatory cytokines, serum cytokines, and pain-related genes in DRG, as well as percentage of DRG CGRP-immunopositive neurons among different groups were compared using non-parametric Kruskal-Wallis test with Dunn’s test as post-hoc comparison. The correlations between paw withdrawal thresholds and different outcome variables were determined using Pearson’s correlation. All statistical analyses were conducted using Prism version 7 (GraphPad Software, Inc., La Jolla, CA) with level of significance set at 0.05.

## Results

### General health

Sham surgery and IVD injury procedures were well-tolerated with no intraoperative complications from daily general physical examination. Among BDPP-treated animals, there were no significant differences in daily BDPP consumption before and after surgery with average consumption of 35.0±6.4 and 38.7±6.4ml of BDPP-infused water per rat per day, respectively. There was also no observable adverse effect following oral BDPP consumption.

**Fig 2:**
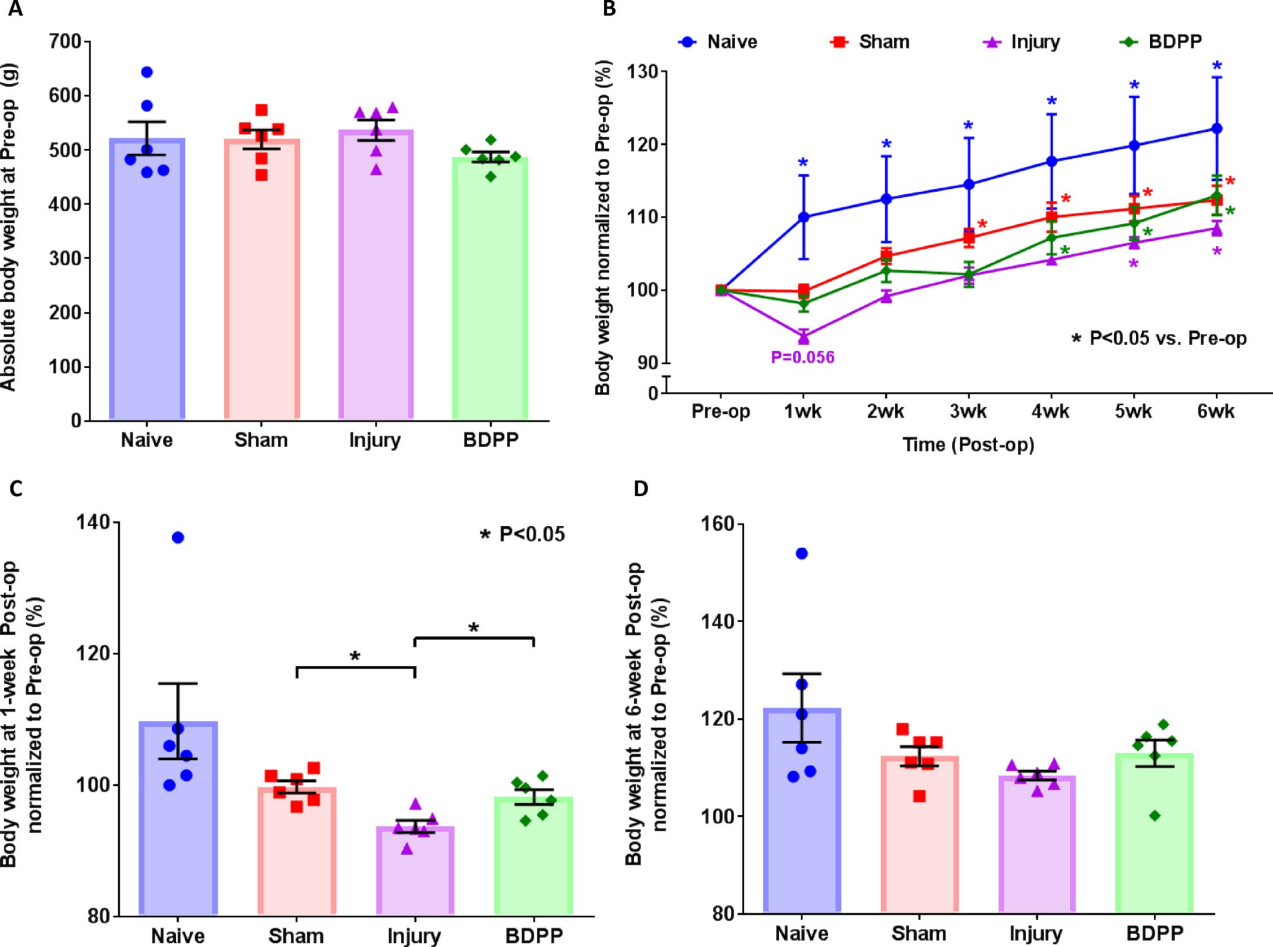
Dietary polyphenols of BDPP alleviated the loss of BW associated with IVD injury. **Animal BWs were monitored weekly across the study**. (A) Baseline (pre-op) BW values obtained 1 week before surgical procedures. Scatter plots presents BW values of individual animals and shows no statistical difference across experimental groups (n = 6 for each group). Cross bars present group mean and SEM BW values. (B) BW values across the duration of the study. Presented are BW values normalized to baseline BW values. Line plots present normalized group mean (± SEM) BW values for each of the experimental groups. * indicates P<0.05 from Pre-op. (C) and (D) Normalized BW values at 1-week & 6-week post-op, respectively. Scatter plots present BW values of individual animals across the experimental groups. Cross bars present group mean and SEM BW values. * indicates P<0.05 between groups.

Animal BWs at baseline were comparable across experimental groups (Fig 2A). BW of Naïve rats increased incrementally and significantly for about 20% throughout the 6-week study (Fig 2B). Both sham surgery and IVD injury interfered with normal BW gain. Animals from both Sham and Injury group maintained lower post-op BWs compared to Naïve animals throughout the study duration (Fig 2B). However, animals in the Injury group exhibiting more severe post-op BW loss and maintained lower BWs compared to Sham animals. Notably, IVD injury-induced weight loss was attenuated by dietary BDPP treatment, and BW of the BDPP animals were comparable to Sham animals throughout the 6 week study period (Fig 2B). BW of Sham animals recovered towards normal levels across the duration of the study and returned to normal BW gain by weeks 3–6 (Fig 2B). However, the IVD-injured rats restored their normal BW gain at 5-week post-op. Lastly, BDPP-treated rats restored their normal BW gain at 4-week post-op, and tended to have higher BW than Injury animals across the duration of the study (Fig 2B–2D). Together, our evidence suggests dietary BDPP may help attenuate the BW loss associating with IVD injury.

### Mechanical allodynia

**Fig 3:**
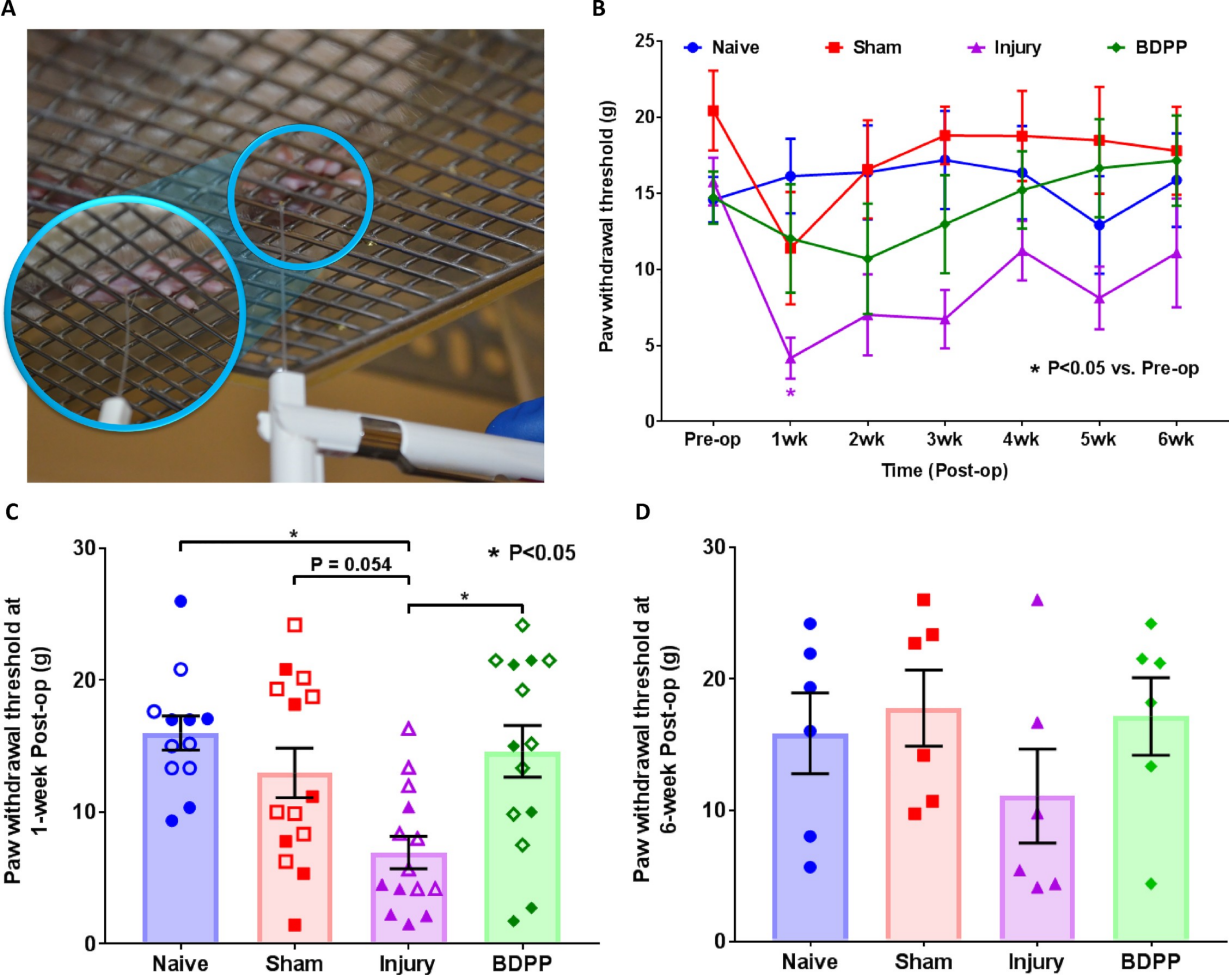
Dietary polyphenols of BDPP alleviated the painful behavior associated with IVD injury. (A) Hindpaw mechanical hyperalgesia was measured using the von Frey test, where a decreased hindpaw withdrawal threshold indicates enhanced sensitivity to pain. (B) Changes of pain sensitivity across the duration of the study. Presented are withdrawal thresholds normalized to baseline (pre-op) values. Line plots present normalized group mean (±SEM) withdrawal threshold values of individual experimental treatment groups. * indicates P<0.05 from Pre-op. (C) and (D) Normalized withdrawal thresholds at 1-week & 6-week post-surgery, respectively. Scatter plots present withdrawal thresholds of individual animals across the experimental groups (n = 12 for Naïve, n = 14 for Sham, n = 14 for Injury & n = 14 for BDPP). Cross bars present group mean and SEM withdrawal thresholds. * indicates P<0.05 between groups.

Mechanical allodynia was measured weekly by applying the von Frey test to rat hindpaws (Fig 3A), where a decreased paw withdrawal threshold indicates enhanced sensitivity to mechanical stimulus.

Naïve animals did not show significant change in withdrawal threshold across the duration of the study (Fig 3B). Sham surgery slightly decreased the withdrawal threshold at 1-week post-op (p>0.05), but withdrawal threshold was restored by week 2 with no statistical difference from pre-op at weeks 2–6 (Fig 3B) or from Naïve animals (Fig 3D). IVD injury resulted in significantly larger reductions in withdrawal threshold at weeks 1–3 post-op (Fig 3B). Besides, the injured rats exhibited significantly lower paw withdrawal threshold than Naïve rats (P<0.05), and also trend to be lower than the sham rats (P = 0.054), which indicate that the IVD injury could induce acute pain behavior in the rats. However, the withdrawal thresholds of animals in the Injury group restored toward normalcy over time (Fig 3B), and were no longer statistically distinct from Sham or Naïve animals at 6-week post-op (Fig 3D). The results indicate that the pain induced by annular puncture with intradiscal PBS injection recovered and did not become a chronic condition.

Dietary DBPP was effective in alleviating pain sensitivity. In particular, the withdrawal threshold of BDPP-treated animals was significantly higher than those from Injury animals, and was comparable to Sham animals in week 1 post-op (Fig 3B and 3C). The threshold maintained consistent across weeks 2–6 with no significant difference from pre-op, and averaged threshold was higher in comparison to Injury animals at 6-week post-op (Fig 3D).

### IVD height

**Fig 4:**
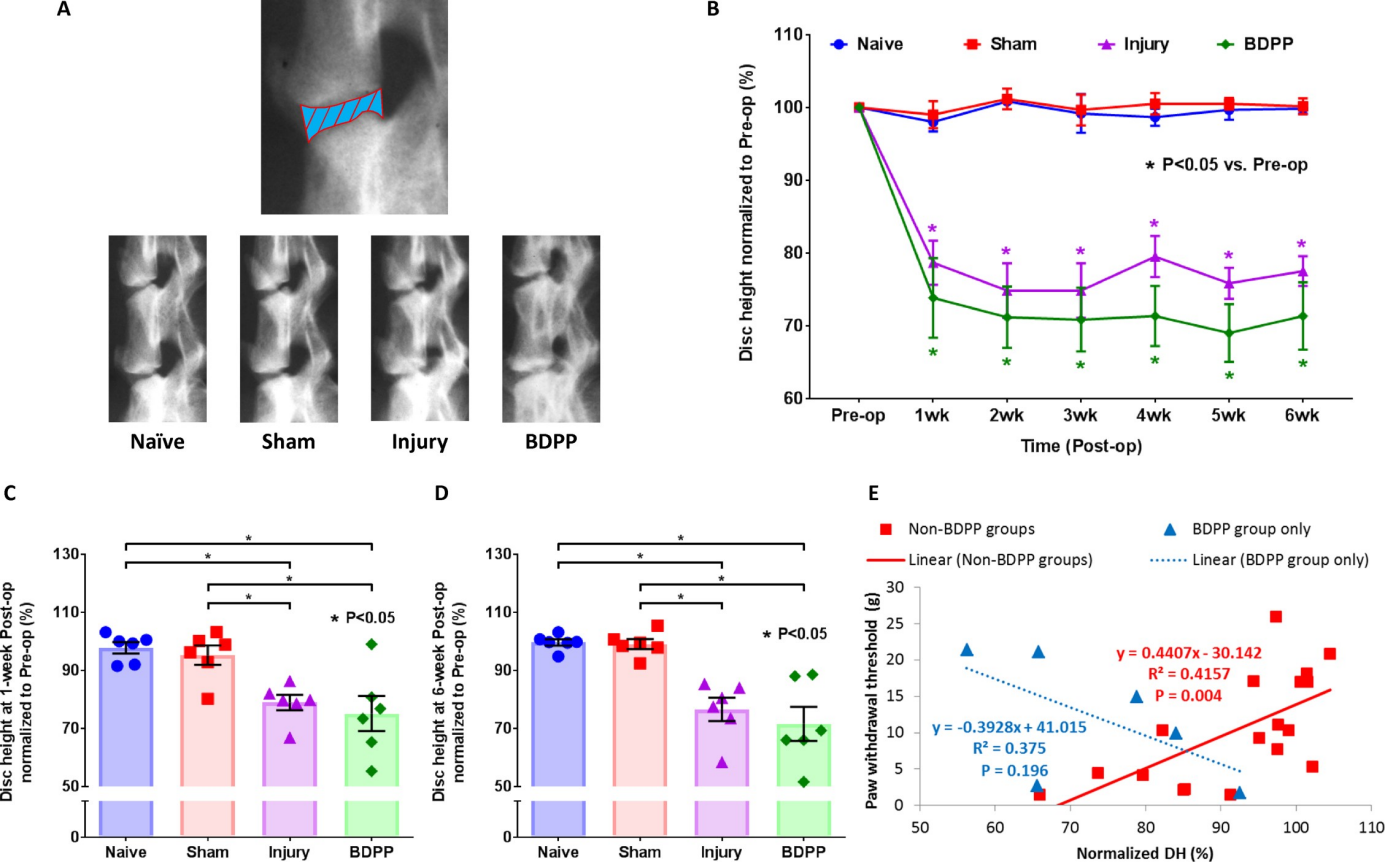
Dietary polyphenols of BDPP did not modulate IVD height loss. (A) Experimental IVD heights (i.e. L3-6 IVDs) were monitored in live animals by radiography. The top radiograph illustrates IVD height measurement by quantifying the averaged distance of intervertebral space between vertebral boundaries (i.e., the blue part with red boundary lines). The bottom radiographs are representative images of rat lumbar spines across all experimental groups. (B) Changes in IVD height across the duration of the study. Line graph presents the normalized IVD height values, i.e. IVD height at each time point normalizing to corresponding group mean values at baseline, as group mean (±SEM) values. * indicates P<0.05 from Pre-op. (C) and (D) Normalized IVD height at 1-week & 6-week after surgical procedures, respectively. Scatter plots presents withdrawal thresholds of individual animals across the experimental groups (n = 6 for each group). Cross bars present group mean and SEM normalized IVD height. * indicates P<0.05 between groups. (E) Pearson’s correlation analysis between paw withdrawal threshold and normalized IVD height (DH) for Non-BDPP groups (i.e. Naïve, Sham and Injury groups) and BDPP group only from the data obtained at 1-week post-op. Scatter plots present data points of individual animal; solid-trendline presents significant correlation; dotted-trendline presents non-significant correlation.

IVD height loss was quantified using lateral radiography (Fig 4A). IVD heights of Sham animals did not show significant changes with time (Fig 4B), and were comparable to and not statistically different from Naïve animals (Fig 4C and 4D), indicating sham surgery had no observable impact on IVD height. However, IVD injury leads to a ~20% loss in IVD height in comparison to pre-op (Fig 4B). This IVD height loss was evident by 1-week post-op and maintained throughout the duration of the study (Fig 4B–4D). The BDPP administration did not modulate the IVD height loss induced by IVD injury (Fig 4B–4D).

Pearson’s correlation analysis showed that mechanical allodynia responses are significantly correlated with IVD height loss (Fig 4E). Interestingly, no significant correlation between mechanical allodynia responses and IVD height was observed among animals in the BDPP group (Fig 4E), indicating that BDPP treatment might disrupt the known association between IVD height and pain.

### IVD morphology

**Fig 5:**
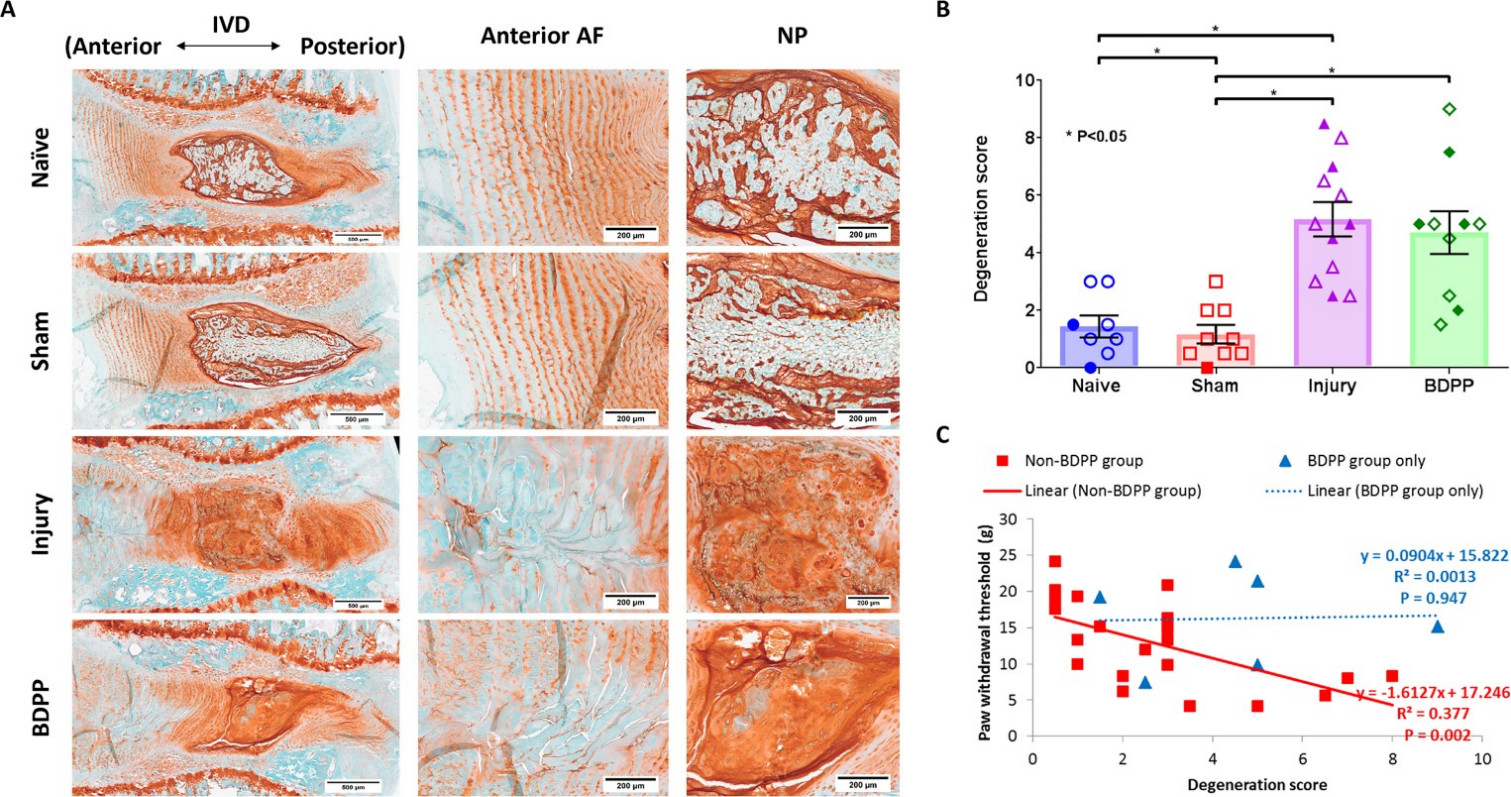
BDPP treatment did not prevent morphological IVD degeneration induced by injury. (A) Representative histological section of IVDs from Naive, Sham, Injury and BDPP groups obtained at 6-week post-op. Scale bar: 500μm. (B) Morphological changes of the designated experimental IVDs represents using a degeneration scale between 0 and 10 (0 = normal and 10 = severe degeneration). Since the degeneration scores of the IVDs from 1-week and 6-week study did not show a significant difference, they were pooled together for statistical analysis. Scatter plots present the degeneration score from individual animals across the experimental groups (n = 8 for Naïve, n = 9 for Sham, n = 12 for Injury & n = 10 for BDPP). Open and solid symbols represent data obtained at weeks 1 and 6 post-op, respectively. Cross bars present group mean±SEM values. * indicates P<0.05 between groups. (C) Pearson’s correlation analysis between paw withdrawal threshold and degeneration score for Non-BDPP groups (i.e. Naïve, Sham and Injury groups) and BDPP group only. Scatter plots present data points of individual animal; solid-trendline presents significant correlation; dotted-trendline presents non-significant correlation.

Normal IVD morphology was observed in both Naive and Sham animals (Fig 5A), and there was no significant difference in degeneration score between the 2 groups (1.44±1.08 and 1.17±0.97, respectively) (Fig 5B). However, IVD degenerative changes, including smaller and more fibrous NP with fewer NP cells, less distinct NP-AF boundary, disorganized AF lamellae, and decreased glycosaminoglycan content and annular cells along the puncture region, were observed in the injured IVD in both Injury and BDPP groups (Fig 5A). Also, the degeneration score of Injury (5.17±2.07) was significantly higher than Naïve and Sham groups, and was comparable to BDPP group (4.7±2.34) (Fig 5B), indicating BDPP administration did not protect against the development of IVD degeneration.

Similar to IVD height, Pearson’s correlation analysis showed that the mechanical allodynia responses are significantly correlated with higher IVD degeneration score (Fig 5C). In contrast, no significant correlation between mechanical allodynia responses and IVD degeneration sore was observed among animals in the BDPP group (Fig 5C), indicating that BDPP treatment might disrupt this association between IVD degeneration and pain.

### Intradiscal pro-inflammatory cytokines

**Fig 6:**
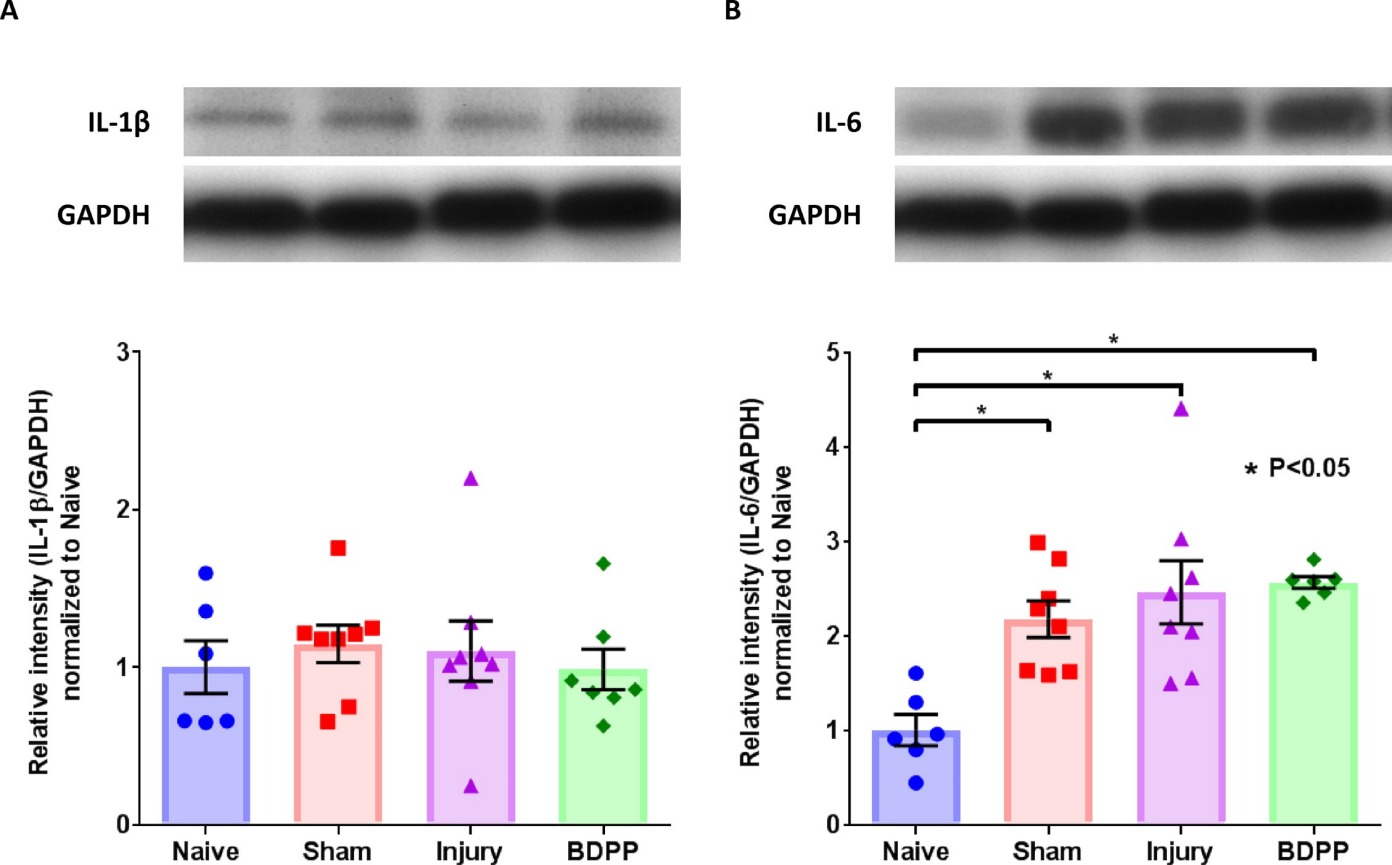
Dietary polyphenols of BDPP did not prevent the upregulation of intradiscal IL-6 associated with surgical procedure or IVD injury. Intradiscal contents of IL-1β and IL-6 at week 1 post-op were assessed by western blot and the intensity of immunostained bands, quantified by densitometry, were used to reflect protein contents. Representative expressions and scatter plots for the protein contents of IL-1β (A), and IL-6 (B) across the experimental groups (n = 6 for Naïve, n = 8 for Sham, n = 8 for Injury & n = 7 for BDPP). Cross bars present group mean±SEM values. * indicates P<0.05 between groups.

Intradiscal IL-1β expression at 1-week post-op had no significant difference across all groups (Fig 6A). However, Sham, Injury and BDPP groups exhibited significantly higher intradiscal IL-6 expression compared to Naïve group at 1-week post-op; the elevated IL-6 expression in Injury and BDPP animals at 1-week post-op was comparable to that observed in the Sham group (Fig 6B). The expressions of intradiscal IL-1β and IL-6 at 6-week post-op as well as TNFα at both 1-week and 6-week post-op were low or undetectable.

### DRG CGRP expression

**Fig 7:**
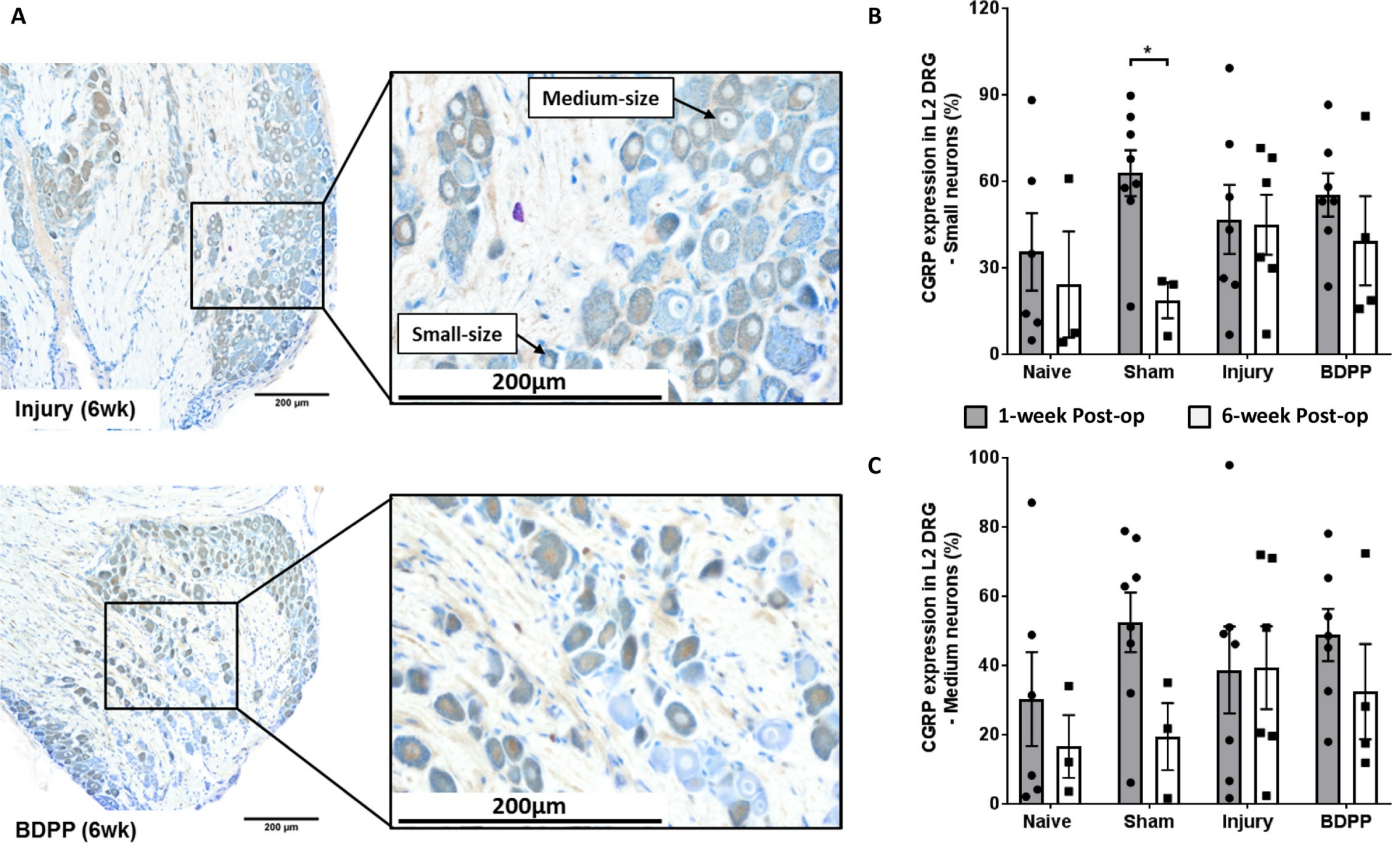
IVD injury and dietary polyphenols of BDPP did not affect the CGRP expression in DRG. (A) Representative L2 DRGs (collected at 6 weeks post-op) with CGRP-immunopositive neurons (stained brown color) from Injury and BDPP groups. Small- and medium-sized neurons, which corresponds to unmyelinated c-fiber and myelinated a-delta fiber, respectively, were determined. (B) and (C) Percentage immunopositivity of CGRP from small- and medium-sized neurons, respectively, at 1-week (n = 6 for Naïve, n = 8 for Sham, n = 7 for Injury & n = 7 for BDPP) and 6-week post-op (n = 3 for Naïve, n = 3 for Sham, n = 6 for Injury & n = 4 for BDPP). Scatter plots present the CGRP immunopositive neurons from individual animals. Cross bars present group mean±SEM values. * indicates P<0.05 between groups.

Percentages of CGRP-immunopositive small- and medium-sized neurons were quantified (Fig 7A). There was no significant difference between groups for both small- and medium-sized neurons (Fig 7B and 7C). Therefore, the DRGs were further analyzed for a list of pain- and inflammatory-related genes using qPCR assays. However, the Sham group exhibited significant downregulation of CGRP expression from 1-week to 6-week post-op (Fig 7B and 7C), which was consistent with the recovery of pain threshold in Sham rats.

### Inflammatory gene expression by qPCR

**Fig 8:**
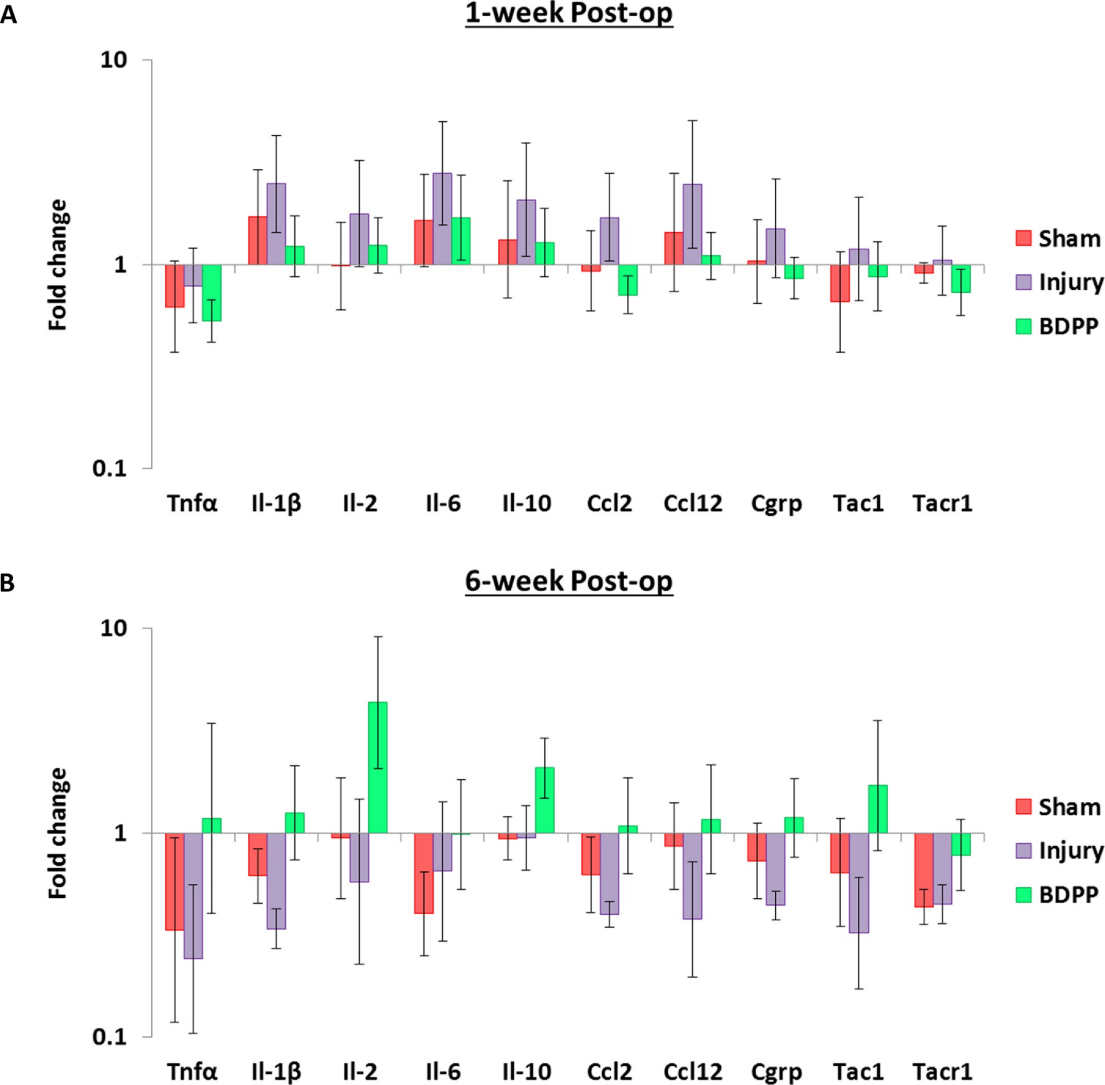
Dietary polyphenols of BDPP trended to modulate the upregulation of pro-inflammatory cytokines and chemoattractants induced by IVD injury in DRGs. Gene expressions (fold change relative to GAPDH and Naïve group) of inflammatory cytokines (tnfα, il-1β, il-2, il-6 and il-10), chemoattractants (ccl2and ccl12), as well as neurotransmitters and receptors (cgrp, tac1 and tacr1) in DRGs collected at (A) 1-week (n = 6 for Naïve, n = 8 for Sham, n = 8 for Injury & n = 7 for BDPP) and (B) 6-week post-op (n = 4 for Naïve, n = 6 for Sham, n = 4 for Injury & n = 6 for BDPP) were determined using real-time qPCR. Cross bars present group mean±SEM values.

Expressions of inflammatory cytokines, chemoattractants, and neurotransmitters and receptors in DRGs isolated at 1-week (n = 6–8 per group) and 6-week (n = 4–6 per group) post-op were quantified. At 1-week post-op, the averaged expressions of il-1β, il-2, il-6, il-10, ccl2, ccl12 and CGRP genes tended to increase in response to IVD injury and this induction tended to be modulated by BDPP treatment (P>0.05, Fig 8A). Although none of these differences was statistically significant, a large number of cytokines/chemokines are simultaneously involved which suggests these changes might synergistically contribute to the development of post-op painful behavior and protection by BDPP.

At 6-week post-op, the averaged expressions of tnfα, il-1β, ccl2, ccl12, and tac1 genes in both Sham and Injury DRGs tended to be downregulated when compared to Naïve DRGs (P>0.05, Fig 8B). On the other hand, expressions of il-2, il-10 and tac1 genes in BDPP-treated DRGs tended to be upregulated when compared to Naïve, Sham or Injury DRGs (P>0.05, Fig 8B). However, it should be noted that none of these observations were statistically significance.

## Discussion

Developing a non-invasive and effective therapy for back pain associated with IVD degeneration remains a high research priority. This study aimed to evaluate whether dietary supplementation with BDPP (a safe and bioactive, polyphenol-rich dietary preparation) was able to alleviate painful IVD degeneration in a previously established rat model [22–24]. We found that dietary BDPP significantly reduced painful behavior and BW loss associated with IVD injury, demonstrating BDPP offers promise as a dietary supplement to help alleviate IVD degeneration-related pain. Notably, dietary BDPP did not lead to any observable improvements in IVD height loss, IVD degeneration, or intradiscal pro-inflammatory cytokine expressions. We also found that the BDPP trended to modulate the upregulation of a large number of pro-inflammatory cytokines and chemoattractants associating with IVD injury in DRGs.

The pathophysiology underlying painful IVD degeneration is complex. In this discogenic pain model, IVD degeneration was induced by annular puncture with saline injection. The annular puncture was a complete annular tear which is known to involve IVD defects, loss of glycosaminoglycan, reduced IVD height, and decreased nuclear pressure, which can increase pain behaviors through increased spinal instability, nerve impingement and irritation, and neural ingrowth and sensitization with increased pro-inflammatory cytokines [42–44]. Consistent with our previous study [23, 24], injured rats exhibited acute and long-term painful behavior with IVD height loss, IVD morphological degenerative changes, elevated intradiscal pro-inflammatory cytokines, as well as upregulated expressions of pain- and inflammatory-related genes and neuropeptide in DRGs. There was no evidence of sustained pain from sham surgery or significantly elevated serum cytokine levels, further indicating that the sustained pain in our discogenic pain model was directly associated with the annular injury and its downstream IVD degeneration processes, which was further supported by our correlation analysis showing significant correlations of pain sensitivity with disc height loss and IVD degeneration score (Figs 4 and 5). Interestingly, the BDPP can alleviate the pain behavior without modulating the IVD degeneration, suggesting that there might be a gap between BDPP in modulating pain and IVD degeneration. Also, the IVD-injured animals treated with BDPP did not exhibit the known statistically significant associations occurring between mechanical allodynia, IVD degeneration score and IVD height loss, suggesting these animals had increased resilience to pain from IVD injury. However, the relatively small sample size for the BDPP-treated rats is a limitation of this study about this phenomenon, and a follow-up study with larger sample sizes is required. Besides, the pain was quantified using von Frey assay to assess the mechanical allodynia in rats in this study. Although this assay has been demonstrated to be the most sensitive measurement for quantifying pain associated with IVD degeneration [22], other pain measurement assays, for example Hargreaves test, open-field test, and grimace scale, should be considered to confirm the IVD-induced back pain in future studies. Besides, discordance between pain and MRI IVD degeneration is known in humans [45], and investigations like this study offer potential to improve our understanding of why some people with IVD degeneration have, or can develop, increased resilience to pain.

The current model of annular injury with single puncture and PBS injection only induced mild to moderate IVD degeneration with temporary upregulation of intradiscal IL-6, but did not induce any longer-term changes or significant change in intradiscal TNFα or IL-1β. Together, the findings suggest this IVD injury is relatively minor, which is consistent with the study by Ulrich and colleagues [46] who found no significant change in pro-inflammatory cytokines levels (IL-1β and IL-8) following single-stab injury, but significantly elevated after triple-stab injury. It is possible that timing also played a role since Miyagi and colleagues [47] found a single annular puncture increased IL-6 levels 4 days following injury, but levels returned to baseline 1-week post-surgery. These findings also contrast our prior studies where annular injury with TNFα injection induced greater degenerative changes, significantly increased intradiscal pro-inflammatory cytokines, and more severe mechanical pain sensitivity [24]. Together results suggest the quantification for the changes of intradiscal pro-inflammatory cytokines depends on severity of injury and time. The relatively minor injury with recovery in mechanical allodynia is a limitation of this study. The PBS injection injury was selected because we expected oral BDPP to have subtle effects compared to localized application, and future investigations are warranted to assess efficacy of oral BDPP in more severe injury models. It should be noted that the intradiscal IL-6 was also elevated in the Sham rats and did not exhibit significant difference from the injured rats. Since the disc was not injured in the Sham rats, the IL-6 might extrinsically penetrate into the disc from surrounding tissues during sham surgery to expose the lumbar spine; although the upregulated IL-6 in injured IVD might result from both intrinsic pro-inflammatory consequences in response to the IVD injury and extrinsic penetration from surrounding tissues. The results suggest that the expression of IL-6 is associated with both surgical procedures and IVD injury.

BDPP is safe for oral consumption, and can be readily applied as a dietary supplement. The dietary polyphenols BDPP consist of grape seed extract, Welch Concord purple grape juice and all trans-resveratrol, and contain distinct and diverse polyphenol compositions. Based on our previous liquid chromatography-mass spectrometry analysis, the grape seed extract is comprised of gallic acid, catechin and epicatechin in monomeric, dimeric, trimeric and oligomeric forms [30] and the Concord grape juice is comprised of a higher diversity of polyphenols [29]. A total of 12 flavonoids (including catechin and epicatechin), 33 anthocyanins, 4 phenolic acids, and 4 proanthocyanidins [29] were identified. BDPP polyphenol with 509 mg/kg BW/day was selected in present study since this dose was well tolerated in rats in our previous study [48]. It should be noted that the Welch grape juice also contains sucrose that was naturally present in the grapes. Therefore, in addition to polyphenols, our BDPP supplementation also provided 3.8 g/Kg BW/day of sucrose from Concord grape juice. The toxicities of grape seed extract and resveratrol have been previously investigated using animal models including toxicology tests, and both studies reported that the orally ingested polyphenols for 90 days did not provide any evidence of toxicity in terms of clinical observations, body weight and food consumption measurements, ophthalmoscopic examinations, hematology, serum chemistry, organ weights, or histopathology [49, 50]. The current study and others assessed body weight as an important indicator for the general health of the animals, which is also one of the important parameters for the clinical observations in the toxicity test [51]. We previously treated mice for 4 [52, 53] to 6 weeks [26] with a dose of 783 mg/kg BW/day BDPP polyphenols. In comparison to control, vehicle-treated mice, we observed no difference in BW gain [26, 52, 53], food consumption [26, 52, 53], water consumption [26, 52, 53] or normal grooming behavior [26]. Based on a drug equivalent dose conversion criterion that takes into consideration body surface area [rat equivalent dose in mg/kg = mouse dose in mg/kg x (mouse wt in kg / rat wt in kg) ^0.33^] [54], the mouse dose is equivalent to a dose of 325.6 mg/kg BW/day BDPP polyphenol for a 500 gm rat. We have also treated normal and diabetic rats for 10 days with 509 mg/kg BW/day BDPP polyphenol (Note that BDPP was referred to as Standardized Grape Polyphenol in that study) [48]. There were no significant in body weight gain in normal and diabetic groups over the course of polyphenol treatment. Together, all findings appear to indicate that BDPP is a safe dietary supplement.

In this study, the BDPP was applied continuously for eight weeks (i.e. started 2 weeks before injury and continued for 6 weeks post-op), and showed that the BDPP would help alleviating BW loss due to spine injury. The changes of BW were comparable with the sham rats and did not negatively affect the general health of rats. Together, the results from previous and our studies revealed that the BDPP is a safe dietary supplement for alleviating discogenic pain, although no comprehensive toxicity tests were performed in this study. Furthermore, non-operative treatments offer much advantage over cell therapies and tissue engineering repairs, since those approaches may involve annular penetration which would induce further IVD degeneration [23, 24, 46, 55, 56], and many novel minimally invasive treatments are still under investigation. Furthermore, BDPP could also be used as an augmentation to additional therapies.

Dietary BDPP shows promising effects to alleviate the IVD degeneration-associated pain in this study. Further development of BDPP for treating annular pain requires a better understanding of BDPP’s mechanisms of action. Polyphenols are generally characterized as anti-oxidant and anti-inflammatory [12, 15]. Recently, select polyphenols (i.e. resveratrol or EGCG) were shown to significantly suppress the productions of pro-inflammatory mediators (i.e. IL-6, IL-8, toll-like receptor, nerve growth factor) and matrix degrading enzymes induced by IL-1β in IVD cells *in-vitro* [18, 19]. We, therefore, hypothesized the dietary BDPP might alleviate the painful behavior by modulating the IVD degeneration and/or intradiscal pro-inflammatory expressions. However, our findings of IVD height, degeneration score, and expressions of intradiscal pro-inflammatory cytokines strongly imply the BDPP has no beneficial effect on IVD degeneration. It is possible that the orally consumed polyphenols from BDPP were not able to penetrate into the IVD due to its avascular nature, and alternate polyphenol formulations with improved IVD penetration might help prevent/slowdown the process of IVD degenerative changes. Previous studies showed that localized intra-articular application of resveratrol or pomegranate at knee joint reduced cartilage tissue destruction, decreased proteoglycan loss in cartilage, and minimized chondrocyte apoptosis induced by surgically-induced osteoarthritis *in-vivo* [57–59], and similar beneficial effects could possibly occur for local application of polyphenols for IVD degeneration [18, 19]. The potential benefits of polyphenols in alleviating IVD warrants further investigations with more severe injuries, and to determine the risks and benefits of treating painful IVD degeneration using chronic dietary supplementation with BDPP as a safe and non-invasive approach versus using more invasive injection of bioactive polyphenols directly to the IVDs. We noted that the promising results of BDPP were demonstrated in male rats in current study. However, males and females have different sex hormones which have been demonstrated to affect the pain behaviors in rat [60, 61]. Different sexes also exhibited different pain pathways [62–64] as well as distinct structural and functional healing responses following IVD injury [65]. Therefore, we believe that sex differences are possible for changes in mechanical allodynia in response to BDPP treatment. However, sex differences were considered beyond the scope of the proposed studies, and warranted for further investigations as an important future direction.

Based on our observation that dietary BDPP did not modulate IVD degeneration, we investigated effects of BDPP treatment on the peripheral nervous system. We analyzed DRG CGRP expression in the DRG neurons since CGRP is a key sensory neurotransmitter associating with neuro-immune communication that plays an important role for the transmission of nociceptive information [66], and was significantly upregulated in DRGs in *in-vivo* IVD degeneration model [31, 67]. Therefore, the changes of CGRP-immunopositive neurons would provide insight about activation and inhibition of peripheral sensitization which could help interpreting the changes of rat pain behaviors in response to IVD injury and BDPP treatment. Interestingly our immunohistochemical analysis indicated the BDPP did not have any significant therapeutic effect on CGRP expression in DRG neurons. In addition to immunohistochemical analysis, we further comprehensively analyzed the expression of pain- and inflammatory-related genes in DRGs. Interestingly, at 1-week post-op, we observed that the gene expressions for all of the DRG cytokines (except tnfα) and chemoattractants monitored in this study tended to increase in response to IVD injury, and this induction tended to be modulated by BDPP treatment. Although these observations are not statistically significant, a large number of cytokines/chemokines are simultaneously involved which suggests the IVD degeneration and intradiscal pro-inflammatory changes resulted from IVD injury could upregulate those DRG cytokines/chemokines which might synergistically contribute to the development of pain behavior in rats, and the anti-inflammatory characteristic of the orally ingested BDPP might mitigate the changes of DRG cytokines/chemokines and pain. The relatively large intra-group variabilities is a limitation of this study, and a follow-up study with larger sample sizes would be required to validate the statistical significance of these observations. On the other hand, we observed downregulated expressions of tnfα, il-1β, ccl2, ccl12, and tac1 genes in both Sham and Injury DRGs as well as upregulated expressions of il-2, il-10 and tac1 genes in BDPP-treated DRGs (P>0.05, Fig 8A and 8B). The changes were not consistent with our hypothesis and the changes of pain behavior. We do not have a clear explanation for this phenomenon, and therefore we suggest further investigating the DRGs using western blot and/or immunohistochemistry to validate the observations.

Previous studies demonstrated that the brain structure and plasticity were modified in patients with chronic back pain and lumbar IVD herniation [68, 69]. Ferruzzi and colleagues [30] and Janle and colleagues [70] showed that orally administered polyphenols could cross blood-brain barrier and accumulate in brain. Moreover, orally consumed BDPP was found to improve brain synaptic plasticity, and showed beneficial effects in cognitive function for Alzheimer’s disease and sleep deprivation-induced contextual memory deficits [16, 25, 26]. Furthermore, accumulated BDPP in brain could downregulate brain pro-inflammatory cytokines and improve brain structure and plasticity, which were reported to be associated with neuropathic and chronic back pain [68, 69, 71–77]. Given that dietary BDPP was effective in modulating pain behavior in our discogenic pain model with absence of observable impacts on IVD and non-significant changes in DRGs, additional studies should focus on exploring the potential contribution of the central nervous system in discogenic pain and the modulation of discogenic pain by BDPP.

Some limitations warrant additional discussion. The volume and method of intradiscal injection were determined from in-vitro and in-vivo pilot studies that refined injection method and titrated the injection volume down to 2.5uL. Briefly, in-vitro pilot studies injected different volumes (2.5, 5 or 10μL) of blue dye into 9 motion segments (L3-4, L4-5 & L5-6) and subsequently loaded on a biomechanical test machine to simulate physiological loading. No leakage was observed for the 2.5μL intradiscal injection while leakage was observed for greater injection volumes. In-vivo pilot studies with 5 animals further determined that 2.5μL volume of PBS injection (using procedures described in this manuscript) did not induce any adverse effects on the rats while injection of higher volumes resulted in dropped foot immediately post-op on physical exam, suggesting nerve irritation from disc bulging due to the high injection volume. Since PBS is clear and we did not use a tracer in the current behavioral studies, we cannot fully reject the possibility that some PBS could have leaked outside of the disc, yet these pilot studies give us high confidence that any potential leakage would be minimal and unlikely to influence results. A micro-injection pump could also be used to control the injection velocity, yet this did not appear to be necessary. Moreover, it should be noted that immunohistochemistry for CGRP was quantified only for small- and medium-sized DRG neurons since they are nociceptive nerve fibers that transmit pain signals [41]. Price [78] determined that about 80% of rat L6 DRG neurons are small- and medium-sized and most neurons are medium-sized. The measurements of small- & medium-sized DRG neurons are therefore believed to be sufficient to quantify the majority of nociceptive changes which are expected to be important in the pain response. Large DRG neurons are mainly myelinated a-beta fibers that generally transmit mechanoreceptive and proprioceptive signals. However, large-sized a-beta fibers have more recently been identified to play a role in pain transmission and allodynia responses [79, 80]. Therefore, the measurement of CGRP immunopositivity in small- and medium-sized neurons is a limitation since large-sized neurons could also play a role in some of the observed changes and warrant characterization in future studies. Furthermore, immunostaining of CGRP had high variability so that western blot analysis is also warranted for future investigations to validate the effects of IVD injury and BDPP on DRGs.

In conclusion, oral consumption of BDPP alleviated pain associated with IVD degeneration induced by annular injury, suggesting dietary BDPP may help alleviating IVD degeneration-related pain. The results of gene expression analysis in DRGs showed that the cytokines and chemoattractants tended to be upregulated in response to IVD injury and the BDPP tended to modulate these changes. Although none of these changes were statistically significant, it should be noted that that a large number of cytokines/chemokines were simultaneously involved. Therefore, results suggest that IVD degeneration and intradiscal pro-inflammatory changes from IVD injury might upregulate DRG cytokines/chemokines which might synergistically contribute to the development of pain behavior in rats, and the anti-inflammatory characteristic of the orally ingested BDPP might mitigate the changes of DRG cytokines/chemokines and pain. As polyphenols have previously been shown to accumulate in brain, it is also possible that the polyphenols could also help mitigate pain development through actions at the level of the central nervous system. Although the mechanisms of BDPP are not fully understood, the promising effect in alleviating pain from IVD injury warrants further investigations with more severe injuries to better understand its potential benefits and mechanism of action of polyphenol-rich preparations for treating back pain.

## Supporting information

S1 Dataset Dataset for the changes of body weight over time from different rats. (PZFX)

S2 Dataset Dataset for the changes of mechanical allodynia over time from different rats. (PZF)

S3 Dataset Dataset for the changes of IVD height over time from different rats. (PZFX)

S4 Dataset Dataset for the IVD degeneration scores from different rats. (PZFX)

S5 Dataset Dataset for the intradiscal pro-inflammatory cytokines from different rats. (PZFX)

S6 Dataset Dataset for the immunopositivity of DRG CGRP expressions from different rats. (PZFX)

S7 Dataset Dataset for the inflammatory gene expressions in DRG from different rats. (RAR)

S1 Raw data Raw data for the intradiscal pro-inflammatory cytokines from different rats. (RAR)
