# Correction: Dietary polyphenols as a safe and novel intervention for modulating pain associated with intervertebral disc degeneration in an *in-vivo* rat model

**DOI:** 10.1371/journal.pone.0225674

**Published:** 2019-11-20

**Authors:** Alon Lai, Lap Ho, Thomas W. Evashwick-Rogler, Hironobu Watanabe, Jonathan Salandra, Beth A. Winkelstein, Damien Laudier, Andrew C. Hecht, Giulio M. Pasinetti, James C. Iatridis

## Notice of Republication

An incorrect version of Figure 1 was published in error. This article was republished on November 8, 2019 to correct for this error. Please download this article again to view the correct version.
